# Development of Policy Recommendations to Support a National Autism Strategy: Case of a Virtual and Inclusive Stakeholder Engagement Process

**DOI:** 10.34172/ijhpm.2022.7182

**Published:** 2022-09-21

**Authors:** Vanessa Tomas, Brittany Finlay, Stephen J. Gentles, Madison Campbell, Daljit Gill-Badesha, Carolyn Abel, Jennifer D. Zwicker, Jonathan Lai

**Affiliations:** ^1^Rehabilitation Sciences Institute, Faculty of Medicine, University of Toronto, Toronto, ON, Canada.; ^2^School of Public Policy, University of Calgary, Calgary, AB, Canada.; ^3^Department of Community Health, Faculty of Human and Social Sciences, Wilfred Laurier University, Waterloo, ON, Canada.; ^4^Faculty of Health Sciences, McMaster University, Hamilton, ON, Canada.; ^5^Faculty of Education, University of British Columbia, Vancouver, BC, Canada.; ^6^Autism Alliance of Canada, Toronto, ON, Canada.; ^7^New York University, New York City, NY, USA.; ^8^School of Public Policy and Faculty of Kinesiology, University of Calgary, Calgary, AB, Canada.; ^9^Autism Alliance of Canada, Ottawa, ON, Canada.; ^10^Institute of Health Policy, Management and Evaluation, University of Toronto, Toronto, ON, Canada.

## Introduction

 Autism is a lifelong neurodevelopmental condition estimated to affect one in 54 people,^1^ whose prevalence is not affected by geography, race, or socioeconomic factors.^2^ Autistic individuals experience barriers to participation in society, and, to address this, Canada has committed to implementing policies aligning with the priorities of the United Nations Convention on the Rights of Persons with Disabilities (UN CRPD).^3^ In line with this, stakeholders have advocated for the development of a Canadian National Autism Strategy (NAS) to address autism policy gaps and organize a coordinated plan of action across sectors.^4^ In response to these calls to action, in December 2019, the Federal Government of Canada announced its commitment to developing and implementing a NAS.^5^

 To support the NAS development process, leadership from the Autism Alliance of Canada (the Alliance; previously known as CASDA) and Kids Brain Health Network (KBHN), developed a policy development fellowship opportunity and selected five trainees to co-lead stakeholder working groups. The aim was to engage a diverse group of stakeholders from across Canada to ensure that policy recommendations were representative of stakeholders’ needs, increasing the applicability and impact.^6,7^ Through meaningful stakeholder engagement, policies can gain legitimacy and support among the communities they aim to serve.^7^ Inclusive engagement in policy development is also stipulated in Article 4 of the UN CRPD, which requires that disabled people and their representative organizations be engaged in decision-making processes that affect their lives.^3^ While citizen engagement in policy development is not a new idea, the application to autism policy development is relatively novel.

 Inclusive engagement in policy development requires a bi-directional flow of information, knowledge, and experiences.^8^ Inclusive engagement processes are flexible and address existing power imbalances to ensure all individuals can meaningfully participate.^9^ In recognition of these ideas, our working groups followed an inclusive and virtual community of practice (CoP) approach. CoP is a social method to engage stakeholders in research, government, and policy-development processes.‎^6,10^ The CoP approach is flexible, focuses on strengthening connections, and supports team members to create a shared understanding and pursue common goals.^‎11^ There has been an uptake in CoP use in health policy development, as it supports meaningful, effective knowledge sharing.^‎12^

 The virtual aspect of our CoP approach permits online communities to form and establish shared priorities, goals and ideas with the potential to mitigate barriers to stakeholder participation (for example, due to geography or health risks), allowing for participation of individuals previously neglected in policy development.^7,13,14^ This, dovetailed with the increase in virtual engagement during the coronavirus disease 2019 (COVID-19) pandemic, suggests that web-based technology may be the future of policy development. However, there is limited literature that delineates the inclusive engagement process in an online context, specifically in policy development. Online communication can be complex, warranting more research and shared knowledge about best practices.^15^

 The purpose of this Viewpoint is to share our process and experiences of applying a virtual CoP approach to the development of stakeholder-informed policy recommendations relevant to a NAS. We first describe the preparation activities that we completed. Second, we outline our experiences and lessons learned from the process of engaging stakeholders in working groups. Finally, we outline our outputs from this process.

## Preparation for Stakeholder Engagement

###  Policy Fellow Selection

 Policy fellows had to have completed or be in a graduate degree program. Fellows were selected based on their experience working in autism/disability-related research fields and expertise in relation to at least one of the Alliance’s five policy pillars (outlined in Figure).

**Figure:**
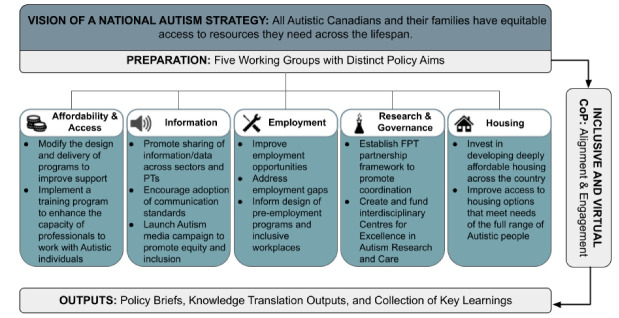
Overview of Working Group Policy Aims and Our Inclusive and Virtual Community of Practice Approach. Abbreviations: PTs, provinces and territories; FPT, federal, provincial and territorial; CoP, community of practice.

###  Policy Fellow Preparation

 Preceding working group engagement, we conducted a preparatory scan of national autism strategies and policies from other countries (Australia, England, Hungary, Malta, New Zealand, Spain, Northern Ireland, Wales, and the United States) to understand the global scope of autism policy and assess applicability to the Canadian context. Findings were synthesized and presented to stakeholders at a webinar in spring 2020, along with an invitation to stakeholders to participate in policy development working groups. The findings from the national scan served as background information and illustrative examples to inform subsequent working group discussions.

###  Working Group Structure

 Leaders of the Alliance and KBHN recruited diverse Canadian stakeholders to participate in working groups via a national call using the Alliance and KBHN listservs and social media. Each working group was co-chaired by a board member from the Alliance and a policy fellow and included five to nine stakeholders. Working group members resided in seven provinces (Alberta, Ontario, British Columbia, Nova Scotia, Saskatchewan, Manitoba, and Quebec) and one territory (Yukon). Members worked in various autism-related organizations in diverse roles and industries (eg, non-profit, for profit, government, service providers) and had different levels of lived experience. Working groups met virtually and monthly from May to September 2020 and had distinct policy aims (Figure).

## Inclusive and Virtual CoP: Our Engagement Process

 The following strategies were used to engage working group members; specifically, how we created alignment and the tangible strategies utilized to support authentic engagement.

###  Alignment

 A key element of our process was to ensure alignment by building a common understanding (conceptual knowledge of process and task-related understanding) between group members and co-chairs, and across working groups. This established a productive engagement process that resulted in a cohesive and impactful final document.

####  Strategies

 As an initial step, we created and distributed a Terms of Reference (ToR) document that outlined working group objectives and decision-making recommendations (Supplementary file 1). This ensured that everyone was aligned in their expectations of the engagement process.

 We also created alignment with respect to the knowledge of working group members, to ensure all members could meaningfully contribute. While we met regularly to discuss strategies for alignment, each group took a different approach to meet the needs of their respective groups. This enhanced the inclusivity of our engagement process, as it enabled us to choose an approach that was suited to the diverse needs of our group members. For some working groups, ensuring common understanding involved educational presentations and discussions to conceptually define the focus area, which was needed to understand the scope of potential policy recommendations. For example, the Information group used a conceptual map distinguishing five possible types of information in the context of autism policy.

 Most groups needed to start with a clear understanding of the available federal policy options and constraints to consider when pitching ideas. Working group members were often able to identify issues that policy could address via their lived experiences; however, there was greater difficulty determining specific policy tools that could address these issues or ways to leverage existing policy solutions. To bridge this knowledge gap, we used many different strategies. For example, in the Affordability and Access group, co-chairs used their expertise to provide feasible policy tools for addressing issues identified by group members, which prompted group member feedback, sparked new ideas, and focused discussions. Members of the Housing group were each assigned policy tools to research to increase knowledge among members and ensure productive discussions.

 Alignment across working groups with respect to outputs was achieved by meeting regularly to discuss the contents of our briefs. This led to consistent language in recommendations, avoiding redundancy, cross-referenced ideas and citations, and complementary brief styles.

####  Learnings and Recommendations

 First, we recommend that future policy development working groups share draft policy recommendations at key intervals throughout the process of engagement to ensure alignment of policy documents. Second, we recommend that future policy development working groups provide members with background information to help educate members on the policy process and lifecycle, policy levers, and constraints as part of early engagement. This may include assessing policy development knowledge within the group to identify gaps and preparing educational materials to address these gaps.

###  Virtual Engagement

####  Strategies

 To ensure inclusive virtual engagement during and between the monthly meetings, we used a variety of strategies. For example, using Zoom web-conferencing software to host monthly meetings, online scheduling platforms to book monthly meetings (eg, Doodle Poll, When2Meet), file storage and collaborative writing platforms (eg, Google Drive) to share meeting documents (ie, meeting minutes, articles, webpages) and collaborate on policy recommendation documents between meetings, online platforms to elicit feedback and set priorities (eg, Survey Monkey), and virtual communication platforms to connect between meetings (eg, Slack). During meetings, Zoom functions such as breakout rooms, chat, and screen-sharing supported active engagement by directing attention to specific documents and tasks, offering different modes for communication, and facilitating small group conversations.

 While similar strategies, such as leveraging Zoom functions and using online scheduling platforms, were used across groups, most strategies differed and were specific to group needs and work-style preferences. For instance, the Employment group co-chairs used Survey Monkey to solicit feedback on and prioritize policy recommendations between meetings. The Research and Governance group co-chairs assigned roles to address key action items between meetings. We met to discuss and share virtual strategies being used and our respective learnings throughout the engagement process.

####  Learnings and Recommendations

 First, despite the diverse methods used to engage stakeholders virtually, we all experienced, to some degree, a lack of continuous engagement between meetings. This demonstrated the importance of virtual synchronous meetings and discussion. We recommend that future working group leaders be more adaptive and responsive to stakeholder working styles, preferred engagement methods and explore other methods and strategies (eg, prompts, reminders, longer synchronous meetings). Additionally, adjusting the ToR to stipulate more detailed expectations, weekly time commitments, and example tasks to ensure members can commit fully to engaging may be helpful.

 Second, while some of us had knowledge and experience leading working groups, we all could have benefitted from receiving more formal training regarding stakeholder engagement, particularly with such experientiallydiverse working group members (eg, priorities, job roles, knowledge) in an online setting. We recommend that future engagement processes ensure that co-chairs receive formal training on virtual engagement with a specific focus on working with diverse stakeholders.

 Finally, we recognized the importance of engaging a diverse group of stakeholders that included autistic self-advocates. While our virtual approach allowed for a high degree of stakeholder diversity, there was a notable gap in self-advocates that address critical intersecting identities (ie, gender, race). We recommend that the inclusion of stakeholders with lived experience who address diverse intersectional identities is prioritized when engaging in policy development.^‎16^ This may be noted under ‘membership’ in the ToR when describing member composition, roles, and perspectives. Additionally, we recommend adding a new section in the ToR that includes guiding principles to support safe, ethical engagement and that addresses potential power imbalances.

## Outputs

 The main outcome of the stakeholder engagement process was a series of 14 policy briefs that summarized policy recommendations from the working groups.^17^ We wrote the policy briefs and shared them with working group members for feedback prior to final publication. Briefs were sent to the Public Health Agency of Canada (Division of Children and Youth, Centre for Health Promotion) and are being considered in the ongoing development of the NAS in Canada. We aligned policy recommendations with Public Health Agency of Canada’s NAS framework of social inclusion, economic inclusion, and interventions, and suggested additional attention to areas of research and governance. We presented the policy recommendations and key learnings at the Alliance Leadership Summit in October 2020 and the KBHN conference in November 2020.

## Conclusion

 Our virtual inclusive engagement approach allowed for meaningful involvement of a broad range of stakeholders to build a growing virtual CoP in Canada within the autism sector. The virtual environment created by the COVID-19 pandemic surfaced the ‘how to’ of virtual and inclusive CoP and key learnings through a real-life case example. The second round of the Alliance-KBHN policy fellowships commenced in spring/summer 2021 and provided the opportunity to further develop this work and continue growing a CoP.

 We hope that others who are interested in policy development may consider taking a virtual CoP approach to benefit from the multifarious strategies that can be used, platforms leveraged, and inclusion of a broader range of stakeholders than can be achieved with an in-person CoP. We encourage others to reflect upon and/or utilize our delineated learnings and recommendations.

## Acknowledgements

 We gratefully acknowledge the contributions from KBHN funded through The Networks of Centers of Excellence Program and from the Autism Alliance of Canada. The authors would also like to thank all working group participants for their insights and contributions during the engagement process.

## Ethical issues

 Not applicable.

## Competing interests

 Authors declare that they have no competing interests.

## Authors’ contributions

 Conception and design: all authors; acquisition of data: VT, BF, SJG, DGB, and CA; analysis and interpretation of data: all authors; drafting of manuscript: all authors; critical revision of manuscript for important intellectual content: all authors; obtaining funding: JDZ and JL; supervision: JDZ and JL.

## Funding

 This work was supported by contributions from the Autism Alliance of Canada and KBHN funded through the Networks of Centers of Excellence Program.

## Supplementary files

Supplementary file 1. Terms of Reference.

## References

[1] Maenner MJ, Shaw KA, Baio J (2020). Prevalence of autism spectrum disorder among children aged 8 years—autism and developmental disabilities monitoring network, 11 sites, United States, 2016. MMWR SurveillSumm.

[2] Elsabbagh M, Divan G, Koh YJ (2012). Global prevalence of autism and other pervasive developmental disorders. Autism Research.

[3] United Nations General Assembly. Convention on the Rights of Persons with Disabilities. United Nations; 2007. https://www.un.org/en/development/desa/population/migration/generalassembly/docs/globalcompact/A_RES_61_106.pdf. Accessed August 19, 2022.

[4] Canadian ASD Alliance. Blueprint for a National Autism Spectrum Disorder Strategy How the federal government can lead. CASDA; 2019. https://www.casda.ca/wp-content/uploads/2019/03/Blueprint-for-a-National-ASD-Strategy.pdf. Accessed July 15, 2021.

[5] National Autism Strategy. Government of Canada. 2021. https://www.canada.ca/en/public-health/services/diseases/autism-spectrum-disorder-asd/national-strategy.html. Updated April 26, 2021. Accessed December 1, 2021.

[6] Embrett M, Liu RH, Aubrecht K, Koval A, Lai J (2021). Thinking together, working apart: Leveraging a community of practice to facilitate productive and meaningful remote collaboration. Int J Health Policy Manag.

[7] Mulvale G, Chodos H, Bartram M, MacKinnon MP, Abud M (2014). Engaging civil society through deliberative dialogue to create the first Mental Health Strategy for Canada: Changing Directions, Changing Lives. Soc Sci Med.

[8] Prince M. Absent Citizens: Disability Politics and Policy in Canada. Toronto: University of Toronto Press; 2009.

[9] Nicolaidis C, Raymaker D, McDonald K (2011). Collaboration strategies in nontraditional community-based participatory research partnerships: lessons from an academic–community partnership with autistic self-advocates. Prog Community Health Partnersh.

[10] Arnstein S (1969). A Ladder of Citizen Participation. J Am Inst Plann.

[11] Li L, Grimshaw J, Nielsen C, Judd M, Coyte P, Graham I. Evolution of Wenger’s concept of community of practice. Implement Sci 2009;4(1). 10.1186/1748-5908-4-11. PMC2654669 19250556

[12] Bertone M, Meessen B, Clarysse G, et al. Assessing communities of practice in health policy: a conceptual framework as a first step towards empirical research. Health Res Policy Sys. 2013;11(39). 10.1186/1478-4505-11-39. PMC3819672 24139662

[13] Jiménez-Zarco AI, González-González I, Saigí-Rubió F, Torrent-Sellens J (2015). The co-learning process in healthcare professionals: Assessing user satisfaction in virtual communities of practice. Comput Human Behav.

[14] Bricout J, Baker PMA, Moon NW, Sharma B (2021). Exploring the smart future of participation: community, inclusivity, and people with disabilities. International Journal of E-Planning Research.

[15] Ale Ebrahim N, Ahmed S, Taha Z (2009). Virtual Teams: A Literature Review. Aust J Basic Appl Sci.

[16] Cascio M, Weiss J, Racine E (2021). Making Autism Research Inclusive by Attending to Intersectionality: a Review of the Research Ethics Literature. Rev J Autism Dev Disord.

[17] Canadian ASD Alliance. Policy Compendium: The Development of a National Autism Strategy through Community and Stakeholder Engagement. CASDA; 2020. https://www.casda.ca/policy-compendium/. Accessed July 15, 2021.

