# Supplementary material for: Development of Policy Recommendations to Support a National Autism Strategy: Case of a Virtual and Inclusive Stakeholder Engagement Process
Source: Int J Health Policy Manag. 2022 Sep 21;12:7182. doi: 10.34172/ijhpm.2022.7182 (PMC10125094; doi:10.34172/ijhpm.2022.7182)
Supplement: Supplementary file 1 — Terms of Reference. [file ijhpm-12-7182-s001.pdf]

Article title: Development of Policy Recommendations to Support a National Autism Strategy: Case of a Virtual and Inclusive Stakeholder Engagement Process

Journal name: International Journal of Health Policy and Management (IJHPM)

Authors' information: Vanessa Tomas[1], Brittany Finlay[2]*, Stephen J. Gentles[3], Madison Campbell[4], Daljit Gill-Badesha[5], Carolyn Abel[6,7], Jennifer D. Zwicker[8], Jonathan Lai[9,10]

[1]Rehabilitation Sciences Institute, Faculty of Medicine, University of Toronto, Toronto, ON, Canada.

[2]School of Public Policy, University of Calgary, Calgary, AB, Canada.

[3]Department of Community Health, Faculty of Human and Social Sciences, Wilfred Laurier University, Waterloo, ON, Canada.

[4]Faculty of Health Sciences, McMaster University, Hamilton, ON, Canada.

[5]Faculty of Education, University of British Columbia, Vancouver, BC, Canada.

[6]Autism Alliance of Canada, Toronto, ON, Canada.

[7]New York University, New York City, NY, USA.

[8]School of Public Policy and Faculty of Kinesiology, University of Calgary, Calgary, AB, Canada.

[9]Autism Alliance of Canada, Ottawa, ON, Canada.

[10]Institute of Health Policy, Management and Evaluation, University of Toronto, Toronto, ON, Canada

(*Corresponding author: brittany.finlay1@ucalgary.ca)

Supplementary file 1. Terms of Reference

Autism Alliance of Canada

Alliance canadienne de l'autisme

Policy Development Working Groups

TERMS OF REFERENCE (Version 09April2020)

Purpose

The Working Group is responsible for providing advice and feedback to the Kids Brain Health Network (KBHN) - Autism Alliance of Canada policy practicum projects in the following ways:

- Contextualizing the policies from international strategies related to a Blueprint area presented by KBHN policy fellows at the Autism Alliance of Canada WAM webinar "Informing Canada's Autism Strategy: Lessons From Across the Globe";
- Identify Canadian policy levers related to the specific Blueprint topic at hand and then reviewing materials compiled and circulated by the KBHN-Autism Alliance of Canada fellows in a timely fashion and providing feedback electronically;
- Providing other advice and input as might be required according to the individual expertise of members;
- Attending five one-hour virtual meetings, one per month, throughout the project – May to September 2020.

Reporting

The Working Group reports and makes recommendations to the KBHN-Autism Alliance of Canada practicum supervisors. All recommendations by the group will be taken into consideration. The Autism Alliance of Canada Board of Directors will approve the final decisions before submitting the policy brief to the federal government.

The Committee shall be co-chaired by a Board Member of Autism Alliance of Canada (or designate) and a KBHN Policy Fellow, supported by Autism Alliance of Canada staff (i.e. Director of Strategy and Operations and/or Executive Coordinator).

Membership

The Working Group will consist of Autism Alliance of Canada members who are representative of professional and consumer groups with expertise in the content and policies for the Blueprint area who have signed up based on their topic preference following the KBHN-Autism Alliance of Canada WAM webinar "Informing Canada's Autism Strategy: Lessons From Across the Globe".

Autism Alliance of Canada

Alliance canadienne de l'autisme

The Director of Strategy and Operations at Autism Alliance of Canada will appoint the members to represent key stakeholders from across the country who have expertise and experience in the content and policies for the Blueprint area (autistics, caregivers, service providers, researchers, policymakers). Additional appointments may be based on the recommendations of the Autism Alliance of Canada board if representation is partial of the sector

Recommendations and Decision-making

Recommendations to Autism Alliance of Canada's Director of Strategy and Operations will be made by consensus.

- It is desirable that recommendations are acceptable to all Working Group members;
- If a consensus in the Working Group cannot be reached on a topic, the KBHN and Autism Alliance of Canada Supervisors will be advised and make a final decision as to the direction.

Meetings

Notice of each meeting will be provided three weeks in advance based on the majority of members' availability as determined by a Doodle poll. Members will be asked to notify the co-chairs of their intentions re attendance prior to the meeting.

Members agree to hold all confidential information in trust and strict confidence and agree that it shall be used only for the purposes required to fulfill obligations related to this Working Group, and shall not be used for any other purpose, or disclosed to any third party. Further, members understand that this undertaking survives the termination of the Working Group.

Length of Term

The project term is May 2020 through Sept 2020.

Resignation and New Members

The committee shall be notified when a member resigns or when a new member joins.

Review of Terms of Reference

The Terms of Reference for this committee shall be presented and voted on at the first meeting and will be subject to approval/amendment by a simple majority.
